# Effect of a WeChat Intervention Based on the Common-Sense Model on Breast Cancer–Related Lymphedema Preventive Behaviors: Quasi-Experimental Study

**DOI:** 10.2196/77255

**Published:** 2026-04-24

**Authors:** Xia Luo, Jing Chen, Mingfang Li, Xinyi Li, Jun Yan

**Affiliations:** 1 School of Nursing, Chongqing Medical University, No.1 Medical College Road, Yuzhong District, Chongqing, 400016, China, 86 17358949687; 2 School of Nursing, Sun Yat-sen University, No.74 Zhong Shan Second Road, Guangzhou, 510078, China, 86 134 1634 7847

**Keywords:** breast cancer–related lymphedema, common-sense model, illness perception, preventive behaviors, WeChat

## Abstract

**Background:**

Breast cancer–related lymphedema is the most prevalent postoperative complication among breast cancer survivors. Although mobile health tools are increasingly used for patient education, evidence supporting their efficacy in lymphedema prevention remains limited.

**Objective:**

This study aimed to evaluate the effectiveness of a WeChat-based intervention grounded in the common-sense model (CSM) in improving preventive behaviors, modifying illness perceptions, and reducing lymphedema incidence among breast cancer survivors and to validate the targets of the intervention.

**Methods:**

This study used a quasi-experimental design. Participants (N=192) were recruited from the breast cancer department of a cancer hospital in Guangzhou, China. The control group (n=98) received routine care. The intervention group (n=94) participated in a 3-month CSM-guided WeChat mini-program (“Nantian e-Care”) delivering tailored educational articles, exercise tutorials, arm circumference monitoring, and real-time nurse consultations. Outcomes, including preventive behaviors, illness perceptions, and lymphedema incidence, were assessed 1, 3, and 6 months post surgery. Generalized estimating equations were used for the analysis.

**Results:**

The intervention group exhibited significant improvements in lifestyle adjustments (Wald *χ*^2^_2_=6.9, *P*=.03) and physical exercise adherence (Wald *χ*^2^_2_=6.9, *P*=.03) compared with the control group. Illness perception, including identity (Wald *χ*^2^_3_=8.1, *P*=.04), timeline cyclical (Wald *χ*^2^_3_=8.5, *P*=.04), personal control (Wald *χ*^2^_3_=9.3, *P*=.03), illness coherence (Wald *χ*^2^_3_=29.8, *P*<.001), and behavioral (Wald *χ*^2^_3_=19.5, *P*<.001) and physical factors (Wald *χ*^2^_3_=24.1, *P*<.001) were markedly enhanced. Mechanistically, skin care improvements were driven by intervention effects, personal control, illness coherence, and behavioral attribution. Lifestyle changes were correlated with intervention and illness coherence. Adherence to physical exercise was not statistically significantly affected by the intervention, although a trend was observed. Critically, the intervention group demonstrated a lower incidence of lymphedema at 6 months (7.50% vs 16.48%, *χ*^2^_1_=3.9, *P*=.048).

**Conclusions:**

The CSM-guided WeChat intervention effectively promoted preventive behaviors, optimized illness perceptions, and reduced lymphedema risk. These findings underscore the value of integrating theory-driven mobile health tools into postoperative care and highlight scalable strategies for chronic disease management in resource-limited settings.

## Introduction

Breast cancer–related lymphedema (BCRL) is a chronic and debilitating condition affecting >20% of survivors [1]. BCRL arises from surgical- or radiotherapy-induced lymphatic damage, leading to chronic tissue swelling, fibrosis, and functional impairment. Patients require long-term or lifelong control and management [2,3], which markedly affects their physical and mental health and quality of life [4]. The currently available treatment methods can only relieve symptoms rather than cure BCRL, underscoring the importance of primary prevention.

Prevention of BCRL focuses on the management of risk factors. It is difficult for patients to change the risk factors related to cancer treatment (eg, lymph node resection and radiotherapy). Nevertheless, risk factors related to personal behaviors (eg, overweight or excessive weight fluctuation, injury, and infection) can be controlled and avoided through self-management strategies [5]. BCRL preventive behaviors refer to recommendations and precautions that target the aforementioned avoidable factors, including skin care, avoidance of compression and injury, lifestyle, and functional exercise [6,7]. Previous studies have confirmed that early preventive behaviors can effectively prevent BCRL [8]. However, adherence to recommended behaviors is suboptimal (<50%) [9], highlighting the need for innovative interventions.

The common-sense model (CSM) of self-regulation provides a theoretical framework for understanding how individuals perceive, interpret, and respond to health threats [10]. At its core, the CSM emphasizes the role of illness perceptions (cognitive and emotional representations of a health condition) in shaping coping strategies and health behaviors. Key dimensions of illness perception include identity (symptoms attributed to the illness), timeline (expected duration), consequences, personal and treatment control, illness coherence (understanding of the condition), and emotional representation. CSM has been extensively applied in chronic disease management, where interventions designed to modify illness perceptions, such as providing structured information, correcting misconceptions, and enhancing personal control beliefs, have demonstrated positive effects on health behaviors and clinical outcomes [11-13]. For example, among patients with myocardial infarction, an illness perception–focused intervention significantly improved lifestyle adherence and reduced cardiac risk factors [13], underscoring the potential of illness perception as a modifiable target for behavior change.

In the context of BCRL, patients’ perceptions of the condition, such as beliefs about its causes, controllability, and potential consequences, may significantly influence their engagement in preventive behaviors [14-16]. Our prior longitudinal study supports this proposition, showing that baseline illness perceptions, particularly personal control and causal attributions, predict both initial levels and trajectories of preventive behaviors in breast cancer survivors [17]. Individuals with inaccurate or incomplete perceptions of BCRL often exhibit reduced preventive awareness and adherence. Therefore, a CSM-guided intervention that systematically targets these perceptions holds promise for improving BCRL prevention. Despite this rationale, no study has explored the effects of CSM-based interventions on BCRL-preventive behaviors among breast cancer survivors.

Traditional education methods are challenging in terms of patient accessibility and engagement. In recent years, the development and popularization of mobile technology have provided new ideas for health education. Mobile health–based self-management interventions (eg, webpages, applications, and social media software) have been proven to effectively promote the education and management of patients with breast cancer and reduce the burden on medical staff [18]. However, these studies have mainly focused on lifestyle improvements [19] or symptom management of BCRL [20], and few studies have targeted BCRL prevention. Considering cost-effectiveness, WeChat mini-programs stand out among various mobile health intervention tools [21]. Therefore, on the basis of the CSM and previous research, our group developed a WeChat mini-program named “Nantian e-Care” to deliver tailored and interactive content, facilitate continuous monitoring, and provide timely feedback, potentially enhancing patient engagement and behavior change [22]. This study aimed to evaluate the effectiveness of this CSM-based WeChat intervention in improving BCRL preventive behaviors, modifying illness perception, and reducing the incidence of BCRL among breast cancer survivors.

## Methods

### Design

This study adopted a quasi-experimental design and was prospectively registered in the Chinese Clinical Trial Registry (ChiCTR2100048798). The study followed the TREND statement checklist (Checklist 1) for the reporting of nonrandomized evaluations. A quasi-experimental sequential cohort design was adopted primarily due to pragmatic constraints in implementing a randomized controlled trial within the clinical workflow, aiming to minimize intergroup contamination of the digital intervention. We acknowledge that this design choice introduces potential threats to internal validity from historical changes or maturation between the recruitment periods. To mitigate these threats, all institutional protocols for routine BCRL education were kept consistent throughout the study duration.

### Study Setting and Sampling

Patients who had undergone surgery for breast cancer were recruited by convenience sampling from the breast cancer department of a cancer hospital in Guangzhou, China, between June 2023 and June 2024. The inclusion criteria were as follows: age ≥18 years, diagnosis of primary breast cancer (stages I-III), first unilateral axillary lymph node dissection, primary school educational level or higher, daily WeChat use, and voluntary participation in the study. The exclusion criteria were as follows: cognitive impairment, other types of cancer, bilateral breast cancer, distant metastasis or recurrence, upper limb disability, and other edema symptoms. Participants who read <50% of the articles or incorrectly answered the quality control items of the questionnaires were excluded from analysis.

Considering the risk of intergroup contamination and the required sample size, patients who underwent breast cancer surgery between June and August 2023 were included in the control group. After the 6-month follow-up of the control group, patients who underwent surgery between April and June 2024 were enrolled in the intervention group.

### Sample Size Calculation

The sample size was estimated a priori on the basis of the primary behavioral outcome. For pragmatic planning, the sample size was calculated using the formula for comparing 2 independent means using a *t* test, which provides a conservative estimate for the subsequent longitudinal analysis [23]: *n*_1_=*n*_2_=$\left(1+\frac{1}{k}\right){\left[\frac{(t_{1-\alpha /2}+t_{1-\beta })\;\sigma }{\delta }\right]}^{2}$. In this formula, k denotes the allocation ratio, defined as n1/n2; t_1−α/2_ denotes the 2‑sided critical value from the standard normal distribution for a given significance level α; t_1−β_ denotes the one‑sided critical value from the standard normal distribution for the desired power (1−β); σ denotes the assumed common standard deviation of the outcome variable in both groups; and δ denotes the clinically meaningful difference between the 2 group means. Based on the study by Li et al [24], the mean total scores for BCRL preventive behaviors were 53.45 (SD 7.64) in the control group and 58.92 (SD 8.20) in the intervention group, indicating a mean difference of 5.47 points. this difference represents an approximate improvement of 9% on the total scale score, which is considered a clinically relevant shift in self-reported preventive practices. Setting *α*=.05 (2-sided) and power (1 – *β*)=.90, the initial calculation yielded 46 patients per group. Accounting for an estimated attrition rate of ≤20%, the final target was set at a minimum of 58 patients per group. The final analyzed sample size exceeded the minimum target due to successful recruitment within the study periods. This increased the statistical power of the study beyond the initial 90% target.

### Interventions

The control group received routine nursing care, including perioperative nursing, early postoperative functional exercise, and discharge guidance. Education related to BCRL prevention included prevention instructions combined with oral and written education, as well as an explanation and demonstration of the methods of and precautions to be taken during a 3-stage functional exercise (stage 1, from the first postoperative day to the day of drainage tube removal; stage 2, from the day of drainage tube removal to the day of stitch removal; and stage 3, after the removal of stitches). At the end of the follow-up period, the control group received all the resources similar to the intervention group.

The intervention group received a 3-month Nantian e-Care mini-program intervention (Figure S1 in Multimedia Appendix 1) in addition to routine nursing. This intervention was designed to support patients across key postoperative phases to foster the adoption and maintenance of preventive behaviors. In the immediate postoperative phase (first month), the focus was on establishing foundational knowledge, such as BCRL symptoms and early-stage exercises, and initiating self-monitoring routines. During the subsequent, intermediate phase (months 2‐3), the content reinforced behavioral risk factor management and prepared patients for long-term self-care, aiming to translate knowledge into sustained practice during the critical prevention window. The intervention components included the following: (1) delivery of BCRL-related educational articles (covering definition, causes, symptoms, course, recurrence, prognosis, treatment, and prevention); (2) provision of functional exercise videos corresponding to 3 postoperative stages, equipped with follow-along, timing, and logging features; (3) regular reminders for arm circumference measurement and data upload; and (4) a consultation channel for patient inquiries (Table S1 in Multimedia Appendix 1). The detailed intervention protocol has been described in a prior publication [22]. The main implementation process was as follows:

1. Evaluation: two to three days after surgery, the researchers explained the purpose and significance of the study to eligible patients, obtained informed consent, added patients’ WeChat accounts, guided them to access the Nantian e-Care mini-program, and collected baseline data on illness perception.
2. Planning: the Nantian e-Care automatically planned the routine reminders of the education articles according to the surgery date of each patient and decided whether to intensify the reminders according to the baseline illness perception. The logic of tailoring was based on the revised Illness Perception Questionnaire (IPQ-R). Patients scoring below or above the predetermined thresholds on key CSM dimensions triggered an intensified intervention protocol. This intensification included an increased frequency of reminders and the delivery of additional, targeted educational content focused on the specific perception domains where scores were suboptimal at baseline. In contrast, the routine reminders included the standardized, automated delivery of all educational articles, scheduled based on the patient’s surgery date (Table S1 in Multimedia Appendix 1).
3. Service: according to the plan, the mini-program automatically delivers articles and functional exercise videos. Patients could also read articles and watch videos in the knowledge module. They were required to perform functional exercises for ≥30 minutes daily. The functional exercise video page was automatically timed after entry, and a cumulative exercise of 30 minutes daily was automatically clocked in. Patients were able to modify their exercise duration according to their actual exercise. An instructional video of arm circumference measurements was also available. Patients were required to measure their arm circumference before discharge and at 1, 3, and 6 months after surgery or when they felt unwell.
4. Evaluation and feedback: one or two quizzes were attached at the end of the article, through which patients could evaluate their learning. Follow-up data on preventive behaviors and illness perception were collected at 1, 3, and 6 months after surgery, and scores and suggestions were automatically generated. Participants could then receive timely feedback. For example, patients should ideally be able to correctly identify all 8 symptoms. If the identity dimension was <8 points, BCRL symptoms were displayed on the feedback interface to help users further consolidate relevant knowledge.
5. Coordination and referral: patients could ask questions at any time in the consultation module. Breast cancer surgeons or nurses and lymphedema specialist nurses served as study consultants to answer questions (eg, breast cancer treatment and side effects, care of surgical wounds or drains, and diagnosis of BCRL). If the patient required treatment or professional assistance, they were advised to visit the relevant clinic.

### Measurements

The general information questionnaire included sociodemographic information (eg, age, body mass index, educational level, and residence) and disease- or treatment-related information (eg, pathological diagnosis, tumor stage, number of lymph node resections, adjuvant therapy, and dominance of the affected arm).

### Primary Outcome Measures

The implementation of BCRL preventive behaviors in breast cancer survivors for nearly 1 month was assessed using the Lymphedema Risk-Management Behaviors Questionnaire [14]. The questionnaire included 4 dimensions— skin care, avoidance of compression and injury, lifestyle, and other matters requiring attention—with a total of 18 items. A 5-point Likert scale was used. The reliability and validity of the questionnaire were good (Cronbach α=0.79; content validity index=0.87). Functional exercise after breast cancer surgery is essential for BCRL prevention, and the above questionnaire includes only 1 item for exercise. Therefore, adherence to the functional exercise was assessed using the physical exercise adherence dimension of the Functional Exercise Adherence Scale [25]. This dimension was scored on a 4-point Likert scale, where higher scores indicated better adherence to physical exercise. The Cronbach α for the physical exercise adherence dimension was 0.90. Although the mini-program offered a clocking feature to aid self-tracking, it was not used as the primary outcome measure because follow-up indicated that patients often exercised independently without activating the timer, after becoming familiar with the routines. Therefore, the self-report scale was used to obtain a standardized measure of overall adherence.

### Secondary Outcome Measures

Illness perception of BCRL among breast cancer survivors was measured using the IPQ-R [26], which includes 3 parts and 9 dimensions. Part 1 was the identity dimension, which covered 8 basic symptoms and signs of BCRL. The number of symptoms that patients considered to be related to BCRL was the identity dimension score. A higher score indicated more comprehensive knowledge of BCRL symptoms. Part 2 consisted of 7 dimensions scored on a 5-point Likert scale. Higher scores on the timeline acute/chronic, timeline cyclical, consequence, and emotional representation dimensions indicated a more negative attitude toward the course, recurrence, prognosis, and risk of BCRL. Higher scores on the personal control and treatment control dimensions indicated greater confidence in the prevention and control of BCRL through patients’ own efforts or medical means. Higher scores on the illness coherence dimension indicated a more comprehensive understanding of BCRL. Part 3 was the causes dimension, which included 5 factors: tumor and treatment, psychological, behavioral, physical, and uncontrollable. The scoring method was the same as for part 2. Higher scores indicated a higher likelihood that the patient thought that the occurrence and development of BCRL were related to this factor. The scale had good reliability and validity. The content validity index values for the identity and causes dimensions were 0.92 and 0.81, respectively; and the Cronbach α for the other 7 dimensions ranged from 0.61 to 0.92 [27].

At 3 and 6 months after surgery, the circumference of both upper limbs was measured at the metacarpophalangeal joints, wrist creases, 10 cm above and below the lateral epicondyle, elbow creases, and armpits. BCRL was defined as the affected side measuring >2 cm larger than the unaffected side at any measurement point (2‐3 cm, 3‐5 cm, and >5 cm indicated mild, moderate, and severe BCRL, respectively), a diagnostic threshold consistent with established guidelines for postoperative monitoring [28].

Investigators added participants’ WeChat accounts after enrollment and reminded them to complete the follow-up questionnaires at 1, 3, and 6 months after surgery. The control group completed electronic or paper-based questionnaires based on their preferences. The intervention group completed the follow-up questionnaires on the WeChat mini-program.

### Data Analysis

SPSS Software (version 25.0; IBM Corp) and R for Windows (version 4.3.1; R Foundation for Statistical Computing) were used for statistical analyses. Statistical significance was set at *P*≤.05. An intention-to-treat analysis was performed. Statistical descriptions were performed using the mean, SD, median, IQR, frequency, and composition ratio. A 2-tailed Student *t* test, ANOVA, *χ*^2^ test, and rank-sum test were used for comparisons between 2 groups. The generalized estimating equation method was used to analyze the effects of the intervention on BCRL preventive behaviors and illness perception. The models included data from all assessment time points: baseline and 1 month, 3 months, and 6 months post surgery. The scores for each dimension were used as dependent variables, and group, time, and the group × time interaction term were included in the model as independent variables. The statistical significance of the group × time interaction effect indicated that the variable scores of the 2 groups changed differently over time, indicating that the intervention was effective [29].

Intervention targets were verified according to the causal step model proposed by Baron and Kenny [30]. Initially, the effects of the interventions on preventive behaviors and illness perception were examined separately. Subsequently, the effects of the intervention and illness perception on preventive behaviors were simultaneously considered. The group, time, and group × time terms and illness perception dimensions (measured at the same follow-up time points as the preventive behaviors) were used as predictive variables, and each dimension of preventive behaviors was used as an outcome variable for the generalized estimating equation analysis, respectively. The statistically nonsignificant effect of illness perception on preventive behaviors indicated that the effect of the intervention on preventive behaviors was unrelated to illness perception. A statistically significant effect of illness perception and a statistically nonsignificant intervention effect on preventive behaviors indicated that the effect of the intervention was mainly caused by changes in illness perception. The statistically significant effects of both intervention and illness perception on preventive behaviors indicated that the effect of the intervention on preventive behaviors was caused by both intervention and illness perception.

### Ethical Considerations

This study was approved by the ethics committee of the Sixth Affiliated Hospital of Sun Yat-sen University (no. L2019ZSLYEC-001). This study was performed in line with the principles of the Declaration of Helsinki and followed the principles of voluntary participation, confidentiality, and no harm. Participants were informed that their participation was entirely voluntary and that they had the right to withdraw from the study at any time without providing a reason and without any negative consequences for their future care or their relationship with the hospital. All participants provided oral or written informed consent for participation and the publication of their anonymized data. At the end of follow-up, the participants received a compensation of ¥30 to ¥50 (US $4.39-7.32) based on their compliance.

JY is the creator and owner of the mini-program and authorizes the use of images in the manuscript.

## Results

### Characteristics of the Sample

**Figure 1.**
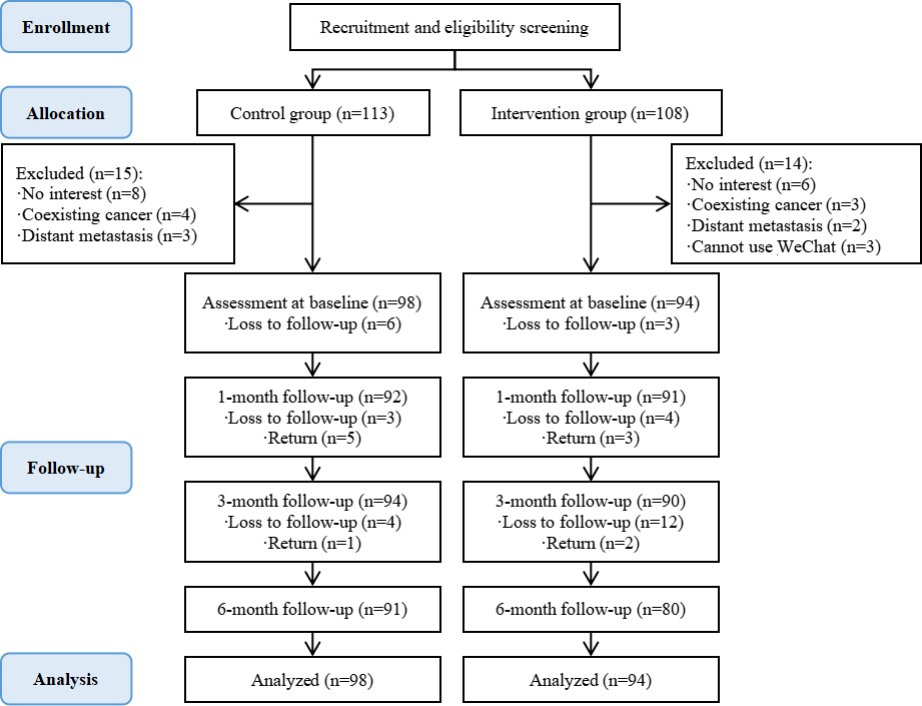
Flow diagram of the participants. “Return” indicates participants who were temporarily lost to follow-up at the previous time point but returned to the study at the current follow-up.

Patient attrition resulted from loss of contact, lack of time or energy, unwillingness to continue participation, or meeting the elimination criteria. The loss-to-follow-up rates were 5.78% ([6 + 4 + 7]/[98 × 3]) in the control group and 7.45% ([3 + 4 + 14]/[94 × 3]) in the intervention group. Following the intention-to-treat principle, all initially allocated participants (N=192; 98 in the control group and 94 in the intervention group) were included in the final analysis, as illustrated in the participant flow diagram (Figure 1).

A total of 192 breast cancer survivors aged 27 to 75 years were analyzed. All participants were women, except for 1 man in the intervention group (Table 1). The baseline characteristics between the 2 groups were not significantly different (*P*>.05; the exact *P* values are reported in Table 1).

**Table 1.** Baseline characteristics of the 2 groups.

Variable	Control group (n=98)	Intervention group (n=94)	*t* test/chi-square/*z* score
Age (years), mean (SD)	47.47 (10.54)	46.56 (9.53)	0.624^a^ (*df^b^*=190, *P*=.53); –0.479^c^ (*P*=.63)
<45, n (%)	43 (43.9)	43 (45.7)	
45‐59, n (%)	41 (41.8)	41 (43.6)	
≥60, n (%)	14 (14.3)	10 (10.7)	
BMI (kg/m^2^), mean (SD)	22.85 (3.06)	22.86 (3.11)	0.660^a^ (*df*=190, *P*=.98); –0.575^c^ (*P*=.56)
<18.5, n (%)	5 (5.1)	9 (9.6)	
18.5‐24.0, n (%)	63 (64.3)	49 (52.1)	
≥24.0, n (%)	30 (30.6)	36 (38.3)	
Educational level, n (%)			–0.324^c^ (*P*=.75)
Primary school	14 (14.3)	8 (8.5)	
Junior high school	17 (17.3)	25 (26.6)	
Senior high school	32 (32.7)	24 (25.5)	
Bachelor’s degree or higher	35 (35.7)	37 (39.4)	
Residence, n (%)			
Urban	66 (67.3)	59 (62.8)	0.4^d^ (*df*=1, *P*=.51)
Rural	32 (32.7)	35 (37.2)	
Tumor stage, n (%)			
I	13 (13.3)	13 (13.8)	–0.007^c^ (*P*=.99)
II	52 (53.0)	49 (52.2)	
III	33 (33.7)	32 (34.0)	
Number of lymph nodes removed, mean (SD)	15.93 (7.65)	16.68 (8.97)	–0.256^a^ (*df*=190, *P*=.53)
Adjuvant therapy received, n (%)			
Chemotherapy	85 (86.7)	77 (81.9)	0.8^d^ (*df*=1, *P*=.36)
Radiotherapy	53 (54.1)	62 (66.0)	2.8^d^ (*df*=1, *P*=.09)
Endocrine therapy	51 (52.0)	40 (42.6)	1.7^d^ (*df*=1, *P*=.19)
Targeted therapy	30 (30.6)	27 (28.7)	0.08^d^ (*df*=1, *P*=.78)
Affected arm that was dominant, n (%)	53 (54.1)	44 (46.8)	1.0^d^ (*df*=1, *P*=.31)
Family history of breast cancer, n (%)	6 (6.1)	4 (4.3)	0.07^e^ (*P*=.80)

a *t* test.

b df: degrees of freedom.

c Mann-Whitney *U* test.

d Pearson *χ*2 test.

e Continuity correction of *χ*2 test.

### Effect of Intervention on Preventive Behaviors

**Figure 2.**
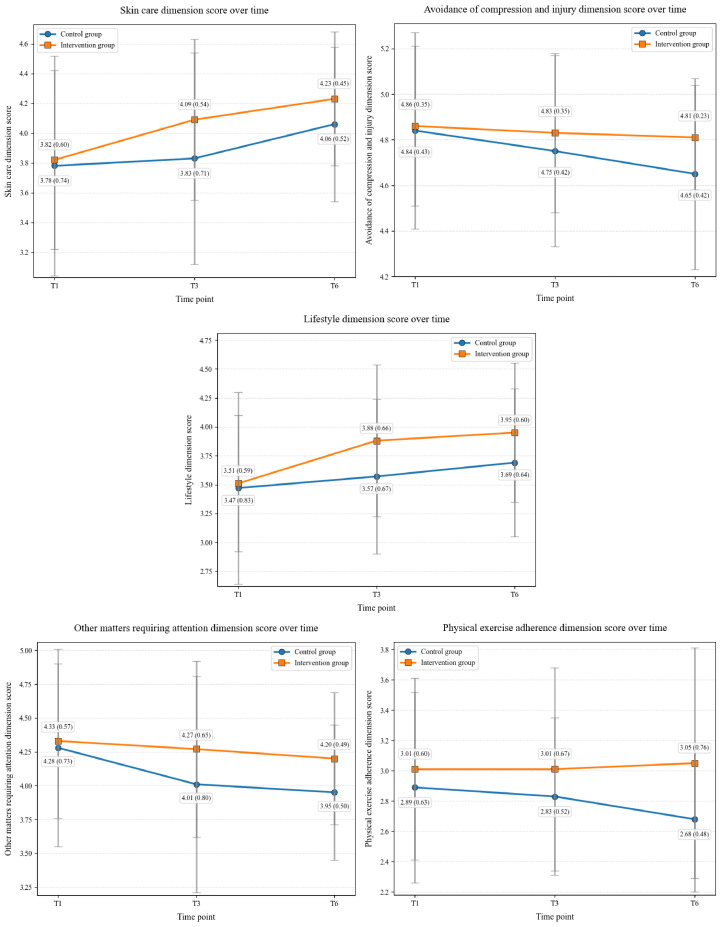
Mean (SD) scores for each dimension of preventive behaviors over time. T1, 1 month after surgery; T3, 3 months after surgery; T6: 6 months after surgery.

The results of the generalized estimating equation analysis showed that the group effects of skin care (Wald *χ*^2^_1_=5.5, *P*=.02), avoidance of compression and injury (Wald *χ*^2^_1_=4.6, *P*=.03), lifestyle (Wald *χ*^2^_1_=6.3, *P*=.01), other matters requiring attention (Wald *χ*^2^_1_=8.8, *P*=.003), and physical exercise adherence (Wald *χ*^2^_1_=9.1, *P*=.003) between the 2 groups were statistically significant. The time effects of skin care (Wald *χ*^2^_2_=43.0, *P*<.001), avoidance of compression and injury (Wald *χ*^2^_2_=11.0, *P*=.004), lifestyle (Wald *χ*^2^_2_=38.6, *P*<.001), and other matters requiring attention (Wald *χ*^2^_2_=18.0, *P*<.001) were also statistically significant. Furthermore, the interaction effects of group and time on lifestyle (Wald *χ*^2^_2_=6.9, *P*=.03) and physical exercise adherence (Wald *χ*^2^_2_=6.9, *P*=.03) between the 2 groups were statistically significant. Figure 2 illustrates the changes in the scores for each dimension of preventive behaviors over time in the 2 groups.

### Effect of Intervention on Illness Perception

The results of the generalized estimating equation analysis showed that the group effects of identity (Wald *χ*^2^_1_=4.331, *P*=.04), timeline acute/chronic (Wald *χ*^2^_1_=9.9, *P*=.002), personal control (Wald *χ*^2^_1_=4.0, *P*=.04), illness coherence (Wald *χ*^2^_1_=20.9, *P*<.001) in the 2 groups were statistically significant. The time effects of identity (Wald *χ*^2^_3_=25.0, *P*<.001), timeline acute/chronic (Wald *χ*^2^_3_=25.6, *P*<.001), timeline cyclical (Wald *χ*^2^_3_=22.2, *P*<.001), personal control (Wald *χ*^2^_3_=23.3, *P*<.001), treatment control (Wald *χ*^2^_3_=34.7, *P*<.001), illness coherence (Wald *χ*^2^_3_=136.8, *P*<.001), and behavioral factors (Wald *χ*^2^_3_=45.5, *P*<.001) and physical factors (Wald *χ*^2^_3_=37.4, *P*<.001) of the causes dimension were statistically significant. The interaction effects of group and time on identity (Wald *χ*^2^_3_=8.1, *P*=.04), timeline cyclical (Wald *χ*^2^_3_=8.5, *P*=.04), personal control (Wald *χ*^2^_3_=9.3, *P*=.03), illness coherence (Wald *χ*^2^_3_=29.8, *P*<.001), and behavioral factors (Wald *χ*^2^_3_=19.5, *P*<.001) and physical factors (Wald *χ*^2^_3_=24.1, *P*<.001) of the causes dimension were also statistically significant. Figure S2 in Multimedia Appendix 1 shows the changes in the scores for each dimension of illness perception over time between the 2 groups.

### Effect of Intervention on the Incidence of Lymphedema

In the control group, 8 of 94 patients (8.51%) and 15 of 91 patients (16.48%) were diagnosed with mild BCRL at 3 and 6 months postoperatively, respectively. In the intervention group, 6 of 90 patients (6.67%) and 6 of 80 patients (7.50%) were diagnosed with mild BCRL at 3 and 6 months post surgery, respectively. The *χ*^2^ test revealed a statistically significant difference in the incidence of BCRL between the 2 groups at 6 months after surgery (*χ*^2^_1_=3.9, *P*=.048).

### Verification of Intervention Targets

As shown in Table 2, the intervention (group × time, Wald *χ*^2^_3_=6.7, *P*=.04) and personal control (Wald *χ*^2^_1_=4.5, *P*=.03), illness coherence (Wald *χ*^2^_1_=10.5, *P*=.001), and behavioral factors (Wald *χ*^2^_1_=4.2, *P*=.04) exerted statistically significant effects on the skin care dimension. These findings indicated that the improvement in skin care–related behaviors was caused by a combination of the intervention and changes in personal control beliefs, illness coherence, and behavioral attribution. The effects of the intervention (group × time, Wald *χ*^2^_3_=6.3, *P*=.04) and illness coherence (Wald *χ*^2^_1_=5.5, *P*=.02) on the lifestyle dimension were statistically significant, indicating that the improvement in lifestyle-related behaviors was driven by both the intervention and illness coherence. Although not statistically significant, a trend was observed suggesting that improvements in physical exercise adherence may be attributable to both the intervention (group × time, Wald *χ*^2^_3_=5.7, *P*=.06) and personal control (Wald *χ*^2^_1_=3.8, *P*=.053).

**Table 2.** Generalized estimating equation models for the validation of intervention targets.

Variable	Lymphedema Risk-Management Behaviors Questionnaire								Functional Exercise Adherence Scale	
	Skincare		Avoidance of compression and injury		Lifestyle		Other matters requiring attention		Physical exercise adherence	
	Wald chi-square	*P* value	Wald chi-square	*P* value	Wald chi-square	*P* value	Wald chi-square	*P* value	Wald chi-square	*P* value
Group (*df*^a^=1)	1.6	.20	1.7	.19	4.1	.04	6.2	.01	4.5	.03
Time (*df*=3)	34.6	<.001	12.9	.002	25.7	<.001	13.0	.002	3.6	.17
Group × time (*df*=3)	6.7	.04	1.4	.49	6.3	.04	3.1	.21	5.7	.06
Illness perception										
Identity (*df*=1)	1.5	.22	0.8	.36	0.9	.35	0.3	.56	0.5	.50
Timeline acute/chronic (*df*=1)	0.3	.58	0.03	.87	0.5	.47	0.09	.77	0.6	.45
Timeline cyclical (*df*=1)	1.1	.30	2.0	.15	2.0	.16	2.13	.14	2.1	.15
Consequence (*df*=1)	1.5	.23	0.5	.50	0.1	.72	0.4	.53	3.1	.08
Personal control (*df*=1)	4.5	.03	0.3	.58	1.6	.20	0.35	.55	3.8	.053
Treatment control (*df*=1)	0.7	.40	0.5	.50	0.8	.38	0.08	.77	0.3	.58
Illness coherence (*df*=1)	10.5	<.001	3.4	.07	5.5	.02	0.18	.67	3.0	.09
Emotional representation (*df*=1)	0.006	.94	0.1	.75	0.8	.37	1.6	.20	0.08	.78
Causes										
Cancer and treatment factors (*df*=1)	0.05	.83	0.08	.77	0.04	.85	0.9	.34	0.3	.57
Psychological factors (*df*=1)	0.9	.34	0.2	.68	0.1	.73	1.4	.24	0.005	.94
Behavioral factors (*df*=1)	4.3	.04	0.06	.80	2.6	.11	3.6	.06	0.02	.88
Physical factors (*df*=1)	0.4	.52	2.1	.15	1.7	.19	0.03	.86	0.007	.93
Uncontrollable factors (*df*=1)	1.1	.30	0.1	.75	0.5	.48	0.4	.54	0.02	.90

a *df*: degrees of freedom.

Exploratory generalized estimating equation analyses suggested that improvements in the skin care dimension were associated with the intervention and with concurrent changes in personal control beliefs, illness coherence, and behavioral attribution (see Table 2). Improvements in lifestyle were associated with the intervention and illness coherence, while improvements in physical exercise adherence were marginally associated with the intervention and personal control. These correlational findings suggest that illness perception may be one pathway for behavior change and warrant formal testing in future studies with dedicated designs. These findings align with the CSM proposition that modifying illness perceptions can facilitate health behavior change.

**Figure 3.**
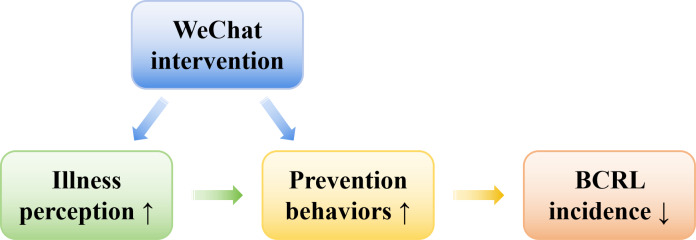
Conceptual framework of the WeChat intervention based on the common-sense model. BCRL: breast cancer–related lymphedema.

Figure 3 presents the conceptual framework of the intervention based on the CSM. The WeChat-based intervention aims to reduce the incidence of BCRL by directly promoting preventive behaviors and/or indirectly improving such behaviors through the modification of illness perceptions.

### Intervention Fidelity and Participant Engagement

Engagement data for the WeChat mini-program are shown in Table S2 in Multimedia Appendix 1. Participants had a median number of logins of 26.5 (IQR 19.0-46.0) and read a median of 40 (IQR 25.8-50.5) articles, exceeding protocol targets. The number of arm circumference measurements (median 3.5, IQR 2.0-5.0) met requirements. However, adherence to daily functional exercise was low (median 2 [IQR 2.0-4.0], 3 [IQR 1.0-10.0], and 4 [IQR 1.0-17.8] days for the 3 phases, respectively) despite the achievement of near-target durations (eg, 30 minutes/day) on days with exercise.

## Discussion

This study demonstrated that the CSM-based WeChat intervention was effective in improving BCRL preventive behaviors and illness perception, as well as in reducing the incidence of BCRL among breast cancer survivors.

### Effects of Intervention on Preventive Behaviors

Consistent with previous results [24], the present findings showed that CSM-based interventions could significantly improve the lifestyle of breast cancer survivors. Taking “maintaining an ideal weight” as an example, patients who underwent surgery might think that the operation was a major trauma and the body required nutritional supplementation, thus taking in excessive energy [31]. During the intervention, the causes of BCRL were presented to patients to raise their awareness regarding the association of overweight or obesity with BCRL risk [32] so that the patients adjusted their diet accordingly.

However, there was no effect of the intervention on behaviors related to skin care, avoidance of compression and injury, or other matters requiring attention. For skin care, the scores showed an upward trend in both groups. Patients could slowly recover their past habits with the gradual healing of the surgical wound and recovery of function of the affected limb even without intervention because these behaviors were consistent with their own habits [33]. However, Li et al [24] showed that a management model based on a smart medical system could effectively improve skin care–related behaviors of breast cancer survivors. The observed difference might be attributed to the differences in the participants included in the 2 studies. Taking “applying lotion” as an example, most patients in this study resided in the Guangdong province or surrounding areas characterized by a humid climate; thus, most of them did not have the habit of applying lotion. However, Li et al [24] recruited participants from northern regions, where the climate is relatively dry; hence, those participants might have had the habit of applying a lotion prior to the disease. It may be more difficult to develop a new habit than to restore an existing habit.

For avoiding compression and injury and other matters requiring attention, scores in both groups showed a downward trend. This is possibly because in the early postoperative period, patients paid more attention to protecting the affected side due to pain caused by the surgical wound. With the healing of the wound and improvement in physical condition, patients might pay less attention in this regard [34], particularly in cases where the behaviors differed from their past habits. In the case of “mopping the floor,” if the affected arm was dominant, the patient was likely to gradually return to previous activity habits [16].

The intervention had an effect on maintenance of patient adherence to functional exercises. In the control group, adherence to functional exercise showed a decreasing trend over time, consistent with the findings reported by Zhang et al [35]. However, there was no significant difference between the scores at the 3 time points in the intervention group. This may be because the articles related to functional exercise in Nantian e-Care made patients more aware of the importance of consistent exercise for BCRL prevention. In addition, the follow-along, timing, and logging features of functional exercises may play a motivating role [36].

### Effects of the Intervention on Illness Perception

The intervention in this study was effective in improving patients’ perceptions of the symptoms and recurrence of BCRL, personal control beliefs, illness coherence, and behavioral and physical attributes. Patients who received education on BCRL prevention were confident that they could prevent BCRL. However, as patients learn more about BCRL, particularly its recurrence and treatment limitations, their confidence may decrease [37]. In this study, personal control beliefs did not improve, possibly because of a ceiling effect [38]. Nonetheless, even though the intervention did not improve patients’ confidence in preventing BCRL, it helped maintain that confidence without a significant decline. Although awareness of BCRL increased over time in both groups, patients in the intervention group received more information, which improved their understanding of BCRL earlier and faster. In addition, the intervention significantly improved the behavioral and physical attributes of BCRL. This may be because the introduction of knowledge about the causes of BCRL provided patients with a deeper understanding of the effects of behavioral and physical factors.

This study found that CSM-based health education could improve the perception of BCRL in breast cancer survivors, consistent with previous studies on patients with chronic diseases [39,40]. Specifically, patients demonstrated increased scores on dimensions such as personal control and illness coherence, reflecting greater confidence in BCRL management and a clearer understanding of BCRL, and reported decreased scores on dimensions such as timeline cyclical and emotional representation, indicating reduced perceptions of recurrence risk and emotional distress. This study suggests that, compared with traditional face-to-face or paper-based education, the intervention combined with WeChat could more effectively improve the delivery of information and patients’ illness perception of BCRL. Li et al [24] also found that afamily nursing network could effectively improve patients’ perceived mastery of their disease knowledge. Patients could effectively obtain information using fragmented time and improve their awareness of breast cancer and BCRL. The integrated management model through a mobile application or WeChat could effectively improve the cognition of patients with breast cancer regarding the causes, symptoms, treatment, and prevention of BCRL [24].

### Effect of Intervention on the Incidence of Lymphedema

In this study, the incidence of BCRL was lower in the intervention group at 6 months after surgery. These results indicated that the postoperative, time-structured intervention could potentially reduce the risk of initial BCRL, consistent with previous studies [24,41]. Notably, in this study, the observed BCRL was mild, and the overall incidence was substantially lower than that reported in previous studies. BCRL is multifactorial and has a low incidence. Therefore, it is unclear whether the effect of our intervention on the risk of BCRL was greater than that reported previously. The risk of developing BCRL increases over time. Further investigation of its occurrence 1 year or more after surgery is warranted to explore the long-term effects of the intervention.

### Verification of Intervention Targets

This study found that some dimensions of illness perception can be identified as intervention targets. In other words, the implementation of preventive behaviors among breast cancer survivors could be improved by increasing their awareness of BCRL, personal control beliefs, and attribution of behavioral factors. This further validates the notion that illness perception influences coping behavior in CSM and provides an evidence-based foundation for future clinical interventions.

Regarding the intervention’s feasibility and acceptability, informal feedback from participants during follow-up indicated that the WeChat mini-program was generally perceived as convenient and user-friendly. Participants appreciated the structured information, the ability to revisit exercise videos, and the timely access to nurse consultations. However, some reported challenges in consistently logging exercise sessions within the app after becoming familiar with the routines, as noted in the engagement data. A systematic qualitative evaluation of user experience, including barriers and facilitators to engagement, is an important next step to optimize the intervention and its implementation in routine care.

### Limitations

This study had some limitations. First, the use of a nonrandomized, sequential cohort design constitutes a major limitation. Although baseline characteristics were balanced and routine care protocols remained consistent, unmeasured historical confounding cannot be ruled out. This could include subtle, undocumented shifts in nursing practices, variations in the emphasis of standard patient education by different staff, or broader institutional changes. While there is no direct evidence that such changes occurred, their potential influence on outcomes remains a possibility. Therefore, the observed effects should be interpreted as preliminary evidence of association and feasibility, and causal attribution requires confirmation in a future randomized controlled trial. Furthermore, the correlational design and multiple statistical tests limit causal inference and increase the risk of type I error. Future research should use targeted, hypothesis-driven designs (eg, with staggered measurement of mediators and outcomes) and more robust statistical methods (eg, path analysis with bootstrapping) to formally test the mediating role of specific illness perception dimensions. Second, the primary outcomes of preventive behaviors were assessed via self-report, which carries the risk of recall and social desirability biases. Although the use of validated scales and consistent measurement time points across both groups aimed to mitigate this risk, the more intensive interaction with the intervention platform may have differentially heightened awareness in the intervention group. Future studies would benefit from objective behavioral data (eg, sensor-based activity tracking) where feasible. Third, this study was conducted at a single cancer center in southern China, and all participants were required to be daily WeChat users to engage with the intervention. This sampling strategy, while necessary for the trial’s feasibility, may limit the generalizability of the findings. It potentially excludes individuals who are older or have lower digital literacy. Future research should aim to include more diverse clinical settings and develop alternative delivery strategies to reach non-WeChat users and ensure equitable access to BCRL prevention programs. Fourth, this study included a follow-up period of only 6 months after surgery, which may be insufficient to demonstrate the effect of the intervention on the BCRL incidence. In future, a longer follow-up period should be considered to investigate the long-term effects of the intervention. Finally, BCRL was assessed via circumferential measurement, which, while clinically valid and guideline-recommended, is less sensitive than volumetric or bioimpedance-based assessments. This may have influenced the recorded incidence rates.

### Conclusions

The CSM-based WeChat intervention program for the prevention of BCRL could directly improve the preventive behaviors and illness perception of breast cancer survivors to a certain extent and reduce the risk of BCRL. Preventive behaviors can also be indirectly improved by changing illness perception (ie, by introducing BCRL-related knowledge and improving personal control beliefs). Interventions based on illness perception allow for more precise targeting of underlying cognitive and emotional factors. In future clinical education, WeChat interventions based on illness perception should be considered to assist breast cancer survivors in preventing BCRL.

## Supplementary material

10.2196/77255 Multimedia Appendix 1 Supplemetary material including a figure depicting user interfaces in the WeChat mini-program “Nantian e-Care”, a table providing a description of the intervention program for preventing breast cancer–related lymphedema, graphs depicting the scores for each dimension of illness perception over time, and a table showing the WeChat mini-program engagement metrics.

10.2196/77255 Checklist 1 TREND statement checklist.

## Abbreviations

BCRL: breast cancer–related lymphedema
CSM: common-sense model
IPQ-R: revised Illness Perception Questionnaire
