# Supplementary material for: Effect of a WeChat Intervention Based on the Common-Sense Model on Breast Cancer–Related Lymphedema Preventive Behaviors: Quasi-Experimental Study
Source: JMIR Form Res. 2026 Apr 24;10:e77255. doi: 10.2196/77255 (PMC13108839; doi:10.2196/77255)
Supplement: Multimedia Appendix 1 [file formative-v10-e77255-s001.docx]

| 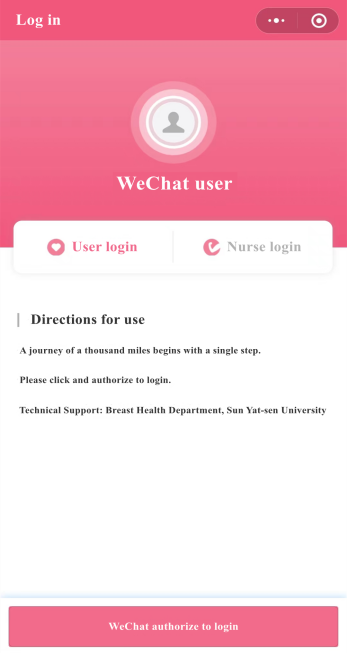 | 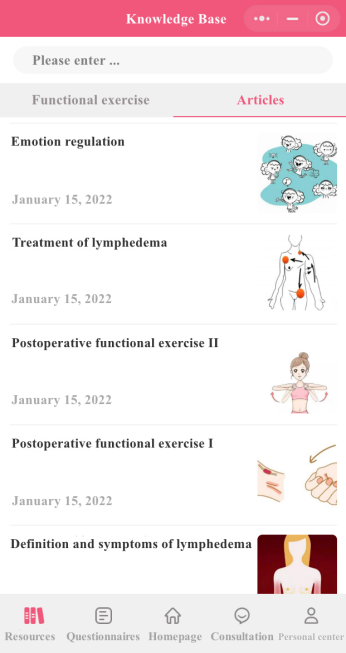 | 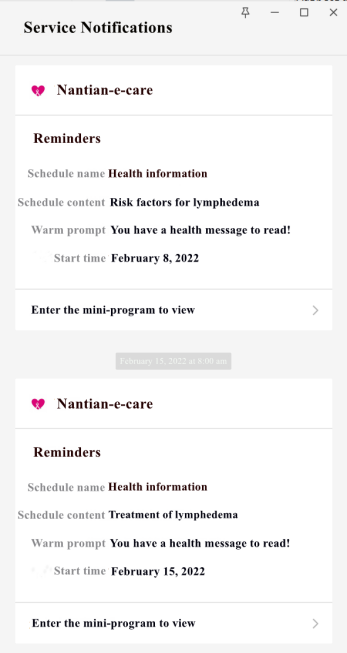 | 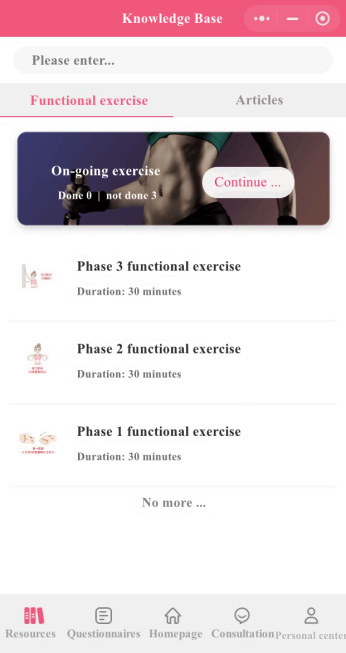 |
| --- | --- | --- | --- |
| 1. Login page | 1. Articles | 1. Service Notification | 1. Functional exercise |
| 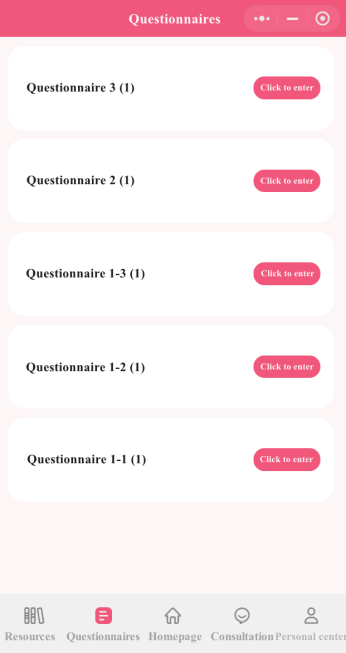 | 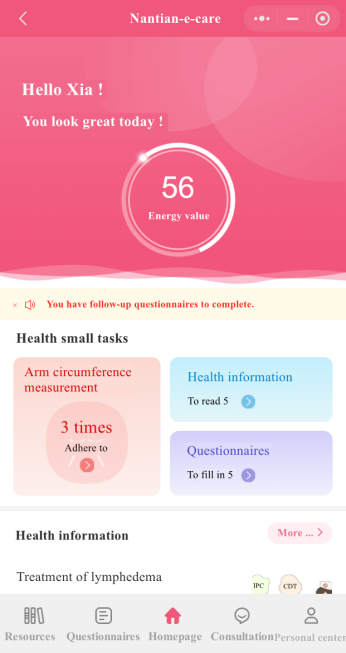 | 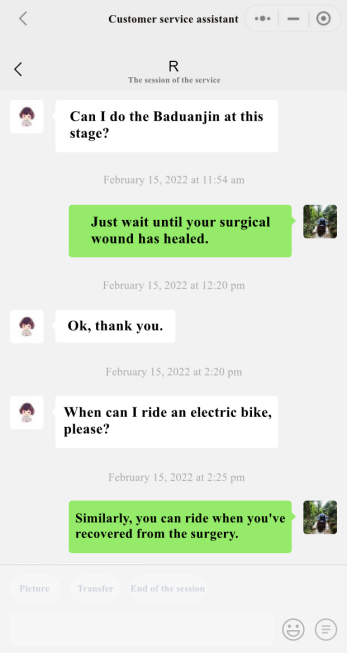 | 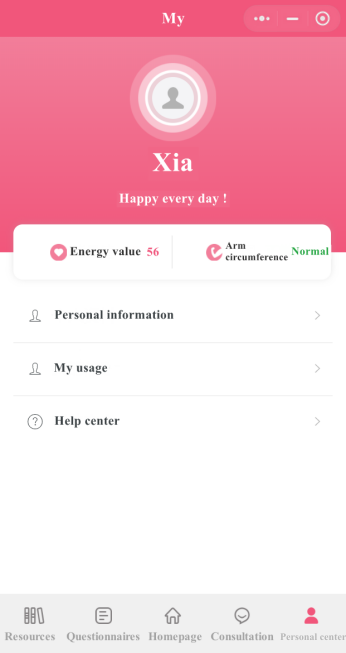 |
| 1. Questionnaires | 1. Homepage | 1. Consultation | 1. Personal center |

**Figure S1. User interfaces in “Nantian e-Care”.**

**Table S1. Intervention program for the prevention of BCRL**

| **Modules** | **Intervention time** | | **Unified intervention** | **Intensive intervention** | **Corresponding theoretical elements ^a^** | |
| --- | --- | --- | --- | --- | --- | --- |
| **Evaluation/**  **Planning** | **2 ~ 3 days after surgery** | | Establish service relationship  Baseline survey and feedback | — | All dimensions of illness perception | |
| **Service** | **1 month**  **after surgery** | **W 1** | Definition and symptoms of BCRL | Identity dimension <8 points | Identity dimension | |
|  |  |  | First stage functional exercise after breast cancer surgery (Article + Video) | Personal control dimension <3 points | Personal control dimension | |
|  |  | **W 2** | Risk factors for BCRL | Behavioral factors <3 points  Physical factors <3 points  Uncontrollable factors >3 points | Causes dimension | |
|  |  |  | Prevention of BCRL  Second stage functional exercise after breast cancer surgery (Article + Video) | Personal control dimension <3 points | Personal control dimension | |
|  |  | **W 3** | Treatment of BCRL | Treatment control dimension >3 points | Treatment control dimension | |
|  |  |  | Course, recurrence and psychosomatic effects of BCRL | — | Timeline acute/chronic, timeline cyclical, and consequence | |
|  |  | **W 4** | Emotion regulation | — | Emotional representation | |
|  |  |  | Third stage functional exercise after breast cancer surgery (Article + Video) | Personal control dimension <3 points | Personal control dimension | |
| **Evaluation/**  **Feedback** |  |  | Follow-up and feedback 1 month after surgery. | | All dimensions of illness perception | |
| **Service** | **2 ~ 3 months after surgery** | **W 5** | Definition and symptoms of BCRL | Identity dimension <8 points | Identity dimension | |
|  |  | **W 6** | Risk factors for BCRL | Behavioral factors <3 points  Physical factors <3 points  Uncontrollable factors >3 points | Causes dimension | |
|  |  | **W 7** | Treatment of BCRL | Treatment control dimension >3 points | Treatment control dimension | |
|  |  | **W 8** | Course and recurrence of BCRL | — | Timeline acute/chronic and timeline cyclical | |
|  |  | **W 9** | Prognosis and impact of BCRL | — | Consequence | |
|  |  | **W 10** | Emotion regulation | — | Emotional representation | |
|  |  | **W 11** | Risk factors for BCRL | Personal control dimension <3 points | Personal control dimension | |
|  |  | **W 12** | Third stage functional exercise after breast cancer surgery (Article + Video) |  |  |  |
| **Evaluation/**  **Feedback** |  |  | Follow-up and feedback 3 months after surgery | | All dimensions of illness perception | |
|  | **6 months after surgery** | | Follow-up and feedback 6 months after surgery | |  |  |
| Note: BCRL: Breast cancer-related lymphedema. ^a^: the common-sense model. | | | | | |  |

| 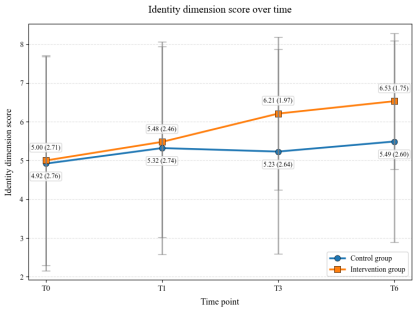 | | 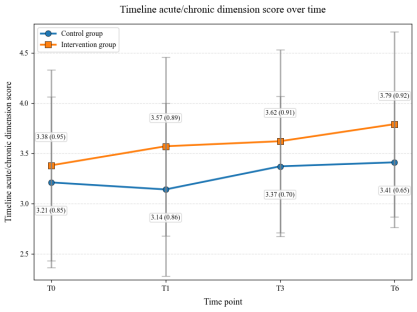 | | 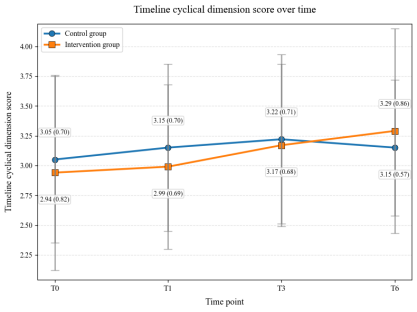 | |  |  |
| --- | --- | --- | --- | --- | --- | --- | --- |
| 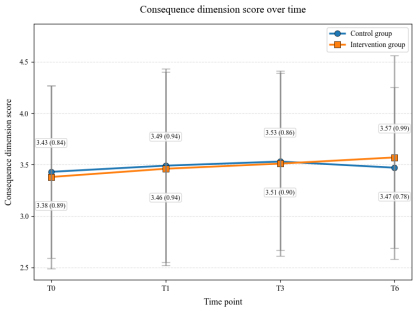 | | 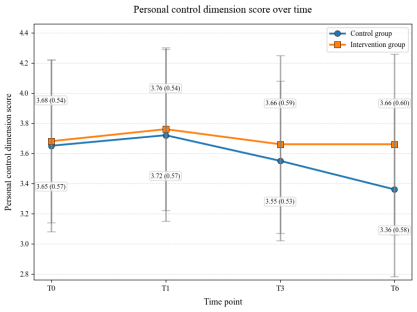 | | 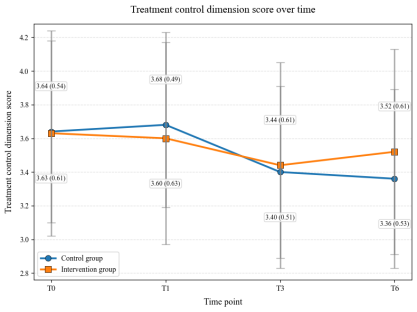 | |  |  |
| 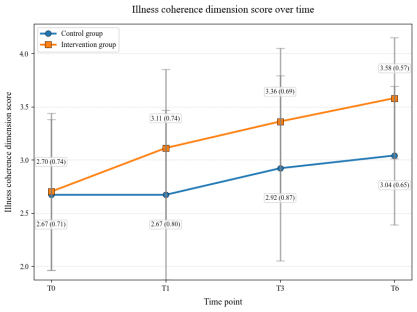 | | 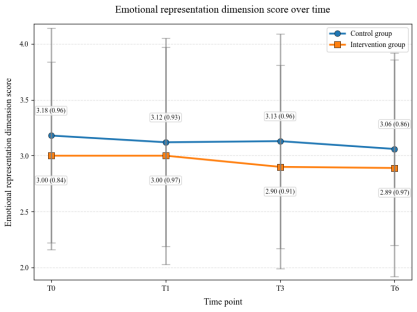 | | 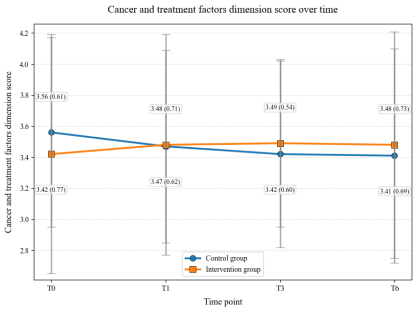 | |  |  |
| 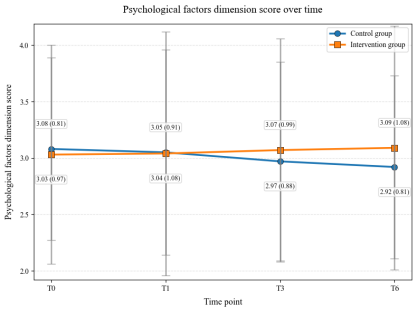 | | 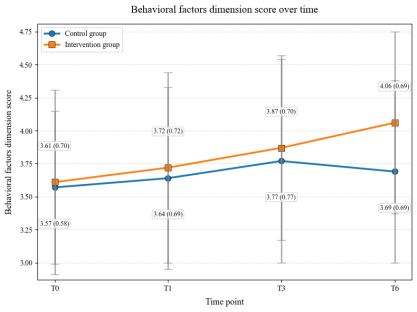 | | 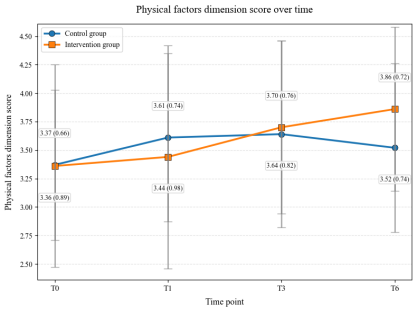 | | 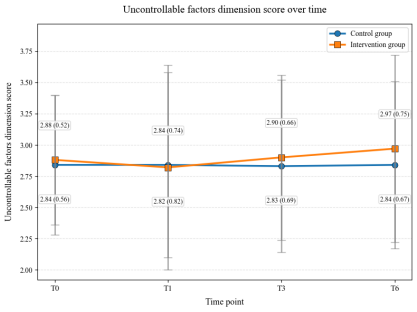 | |

**Figure S2. Scores for each dimension of illness perception over time.**

Note: T1: one month after surgery; T3: three months after surgery; T6: six months after surgery.

**Table S2. WeChat Mini-Program Engagement Metrics (Intervention Group)**

| **Metric** | | ***n*** | **Min** | **Max** | **Median** | **IQR** |
| --- | --- | --- | --- | --- | --- | --- |
| Logins | | 94 | 6 | 190 | 26.5 | (19.00-46.00) |
| Article Reads | | 94 | 6 | 260 | 40 | (25.75-50.50) |
| Arm Circumference Measurements | | 94 | 0 | 24 | 3.5 | (2.00-5.00) |
| Phase1 Functional Exercise | Days | 55 | 1 | 88 | 2 | (1.00-4.00) |
|  | Duration/d (min) | 55 | 3 | 38.5 | 17 | (6.00-30.00) |
| Phase2 Functional Exercise | Days | 52 | 0 | 93 | 3 | (1.00-10.00) |
|  | Duration/d (min) | 52 | 0 | 55.5 | 30 | (17.00-30.00) |
| Phase3 Functional Exercise | Days | 68 | 1 | 152 | 4 | (1.00-16.75) |
|  | Duration/d (min) | 68 | 4 | 55 | 30 | (28.04-30.13) |
| Total Functional Exercise | Days | 78 | 1 | 303 | 6 | (3.00-29.25) |
|  | Duration/d (min) | 78 | 4 | 55 | 28.35 | (19.09-30.00) |

Note: IQR: interquartile range
